# Supplementary material for: Antagonistic Bacteria Bacillus velezensis VB7 Possess Nematicidal Action and Induce an Immune Response to Suppress the Infection of Root-Knot Nematode (RKN) in Tomato
Source: Genes (Basel). 2023 Jun 25;14(7):1335. doi: 10.3390/genes14071335 (PMC10378951; doi:10.3390/genes14071335)
Supplement: Supplementary file 1 [file genes-14-01335-s001.zip › genes-2423557-supplementary.pdf]

Table S1 Bioefficacy of cultural filtrates of bacterial endophytes on hatching of the egg at different concentrations

S. No	Treatments	Number of eggs hatched after an exposure period of *															
		50% (1:1)				25% (1:5)				12.5% (1:10)				7.5% (1:15)			
		**24 h**	**48 h**	**72 h**	**96 h**	**24 h**	**48 h**	**72 h**	**96 h**	**24 h**	**48 h**	**72 h**	**96 h**	**24 h**	**48 h**	**72 h**	**96 h**
1	Bv	0^{a} (1.00)	2.66^{a} (1.64)	7.33^{a} (2.57)	18.33^{a} (4.27)	3.33^{a} (1.77)	9.33^{a} (2.80)	14.6^{a} (3.80)	25.33^{a} (5.02)	7.66^{a} (2.68)	14.33^{a} (3.77)	26.66^{a} (5.15)	31.33^{a} (5.59)	13.33^{a} (3.64)	20.66^{a} (4.53)	34.33^{a} (5.85)	42.33^{a} (6.50)
2	Bh	11.66^{bc} (3.54)	19.33^{bc} (4.43)	29.33^{bc} (5.40)	36.66^{c} (6.04)	16.33^{b} (4.07)	23.33^{bc} (4.79)	32.33^{bc} (5.66)	41.33^{b} (6.42)	27.33^{bc} (5.21)	36.66^{bc} (6.02)	41.33^{bc} (6.40)	48.33^{b} (6.93)	33.67^{bc} (5.80)	41.66^{bc} (6.44)	50.66^{cb} (7.11)	61.66^{c} (7.85)
3	T3	18.67^{de} (4.41)	30.33^{d} (5.54)	41.33^{d} (6.42)	51.00^{d} (7.12)	22.33^{b} (4.73)	34.66^{dc} (5.87)	46.33^{d} (6.7)	59.33^{cd} (7.69)	45.66^{ed} (6.74)	51.33^{de} (7.15)	58.33^{de} (7.63)	64.33^{cd} (8.01)	48.67^{de} (6.94)	56.33^{de} (7.48)	64.66^{de} (8.03)	24.67^{d} (8.63)
4	T4	14.33^{cd} (3.90)	25.33^{cd} (5.07)	34.66^{cd} (5.88)	47.33^{d} (6.87)	19.66^{b} (4.48)	27.33^{bdc} (5.22)	39.33^{cd} (6.27)	54.66^{c} (7.38)	36.66^{cd} (6.04)	44.33^{dc} (6.65)	49.33^{cd} (7.02)	58.67^{c} (7.65)	41.67^{cd} (6.44)	48.33^{dc} (6.94)	57.33^{cd} (7.56)	68.33^{cd} (8.25)
5	T5	7.33^{b} (2.84)	13.66^{b} (3.70)	22.33^{b} (4.69)	27.66^{b} (5.24)	14.33^{b} (3.83)	19.33^{b} (4.39)	26.33^{b} (5.12)	30.66^{a} (5.33)	21.66^{b} (4.64)	29.66^{b} (5.42)	33.67^{ab} (5.78)	37.67^{a} (6.12)	28.00^{b} (5.28)	35.33^{b} (5.93)	42.33^{ab} (6.48)	51.66^{b} (7.17)
6	T6	21.33^{e} (4.71)	35.66^{d} (5.99)	54.33^{e} (7.36)	63.66^{e} (7.97)	27.33^{b} (4.18)	40.33^{d} (6.33)	58.66^{e} (7.65)	65.66^{d} (8.10)	3.66^{ed} (6.93)	13.00^{e} (3.58)	61.33^{e} (7.81)	72.66^{d} (8.52)	53.33^{e} (7.29)	61.66^{e} (7.84)	69.67^{e} (8.34)	78.33^{d} (8.85)
7	Water (Control)	77.66^{g} (8.86)	109.33^{f} (10.47)	168.33^{g} (12.97)	183.33^{g} (13.53)	77.66^{d} (8.83)	109.33^{f} (10.44)	168.33^{g} (12.97)	183.33^{f} (13.53)	77.66^{f} (8.80)	31.67^{g} (5.62)	168.33^{g} (12.97)	183.33^{f} (13.53)	77.67^{f} (8.80)	109.33^{f} (10.44)	168.33^{g} (12.97)	183.33^{f} (13.53)
8	Broth (Control)	54.66^{f} (7.44)	78.33^{e} (8.87)	123.33^{f} (11.09)	146.66^{f} (12.11)	54.66^{c} (7.41)	78.33^{e} (8.85)	123.33^{f} (11.09)	146.66^{e} (12.11)	54.66^{e} (7.37)	18.67^{f} (4.32)	123.33^{f} (11.09)	146.66^{e} (12.11)	54.67^{e} (7.37)	78.33^{g} (8.85)	123.33^{f} (11.09)	146.66^{e} (12.11)
	CD	8.48	0.95	0.91	0.63	1.41	1.16	0.74	0.53	1.01	0.64	0.784	0.577	0.795	0.74	0.69	0.61
	SED	4.00	0.43	0.46	0.31	0-68	0.51	0.36	0.27	0.48	0.31	0.32	0.23	0.34	0.27	0.31	0.28

T_1 – *B. velezensis* VB7, T_2 - *B. hyensii*, T_3 – *B. velezensis*, T_4 – *B. subtilis,* T_5- *B. licheniformis*, T_6- *B.subtilis* T_7- LB broth (Control), T_8- Water (Control)

Figures in parentheses are square root transformed values

*Values are the mean of three replications

Table S2 Bio efficacy of cultural filtrates of bacterial endophytes on mortality of juveniles at different concentrations

S. No	Treat ments	Number of juveniles dead after an exposure period of *															
		**50% (1:1)**				**25% (1:5)**				**12.5% (1:10)**				**7.5% (1:15)**			
		**24 h**	**48 h**	**72 h**	**96 h**	**24 h**	**48 h**	**72 h**	**96 h**	**24 h**	**48 h**	**72 h**	**96 h**	**24 h**	**48 h**	**72 h**	**96 h**
1	T1	33.33^{a} (5.80)	45.31^{a} (6.76)	68.66^{a} (8.31)	81.67^{a} (8.98)	28.31^{a} (5.35)	37.33^{a} (6.15)	64.31^{a} (8.04)	73.65^{a} (8.61)	24.31^{a} (4.98)	32.67^{a} (5.75)	57.33^{a} (7.60)	65.66^{a} (8.13)	19.33^{a} (4.44)	26.67^{a} (5.20)	43.67^{a} (6.64)	51.67^{a} (7.21)
2	T2	26.67^{bc} (5.21)	35.32^{bc} (5.98)	59.33^{bc} (7.73)	69.31^{ab} (8.35)	21.32^{b} (4.65)	31.32^{abc} (5.62)	55.32^{bc} (7.46)	65.32^{bc} (8.11)	17.32^{bc} (4.21)	26.66^{abc} (5.20)	49.67^{ab} (7.08)	58.67^{abc} (7.69)	14.32^{bc} (3.84)	21.32^{ab} (4.67)	36.32^{abc} (6.06)	46.66^{ab} (6.86)
3	T3	19.67^{de} (4.48)	28.31^{cd} (5.36)	51.31^{d} (7.19)	59.32^{cd} (7.73)	15.67^{cd} (3.40)	25.31^{cd} (5.07)	49.32^{cd} (7.05)	59.31^{cd} (7.72)	11.67^{de} (3.48)	19.32^{cd} (4.41)	41.66^{cd} (6.49)	51.66^{cd} (7.22)	9.00^{de} (3.07)	14.67^{cd} (3.87)	29.31^{c} (5.46)	39.32^{bc} (6.30)
4	T4	23.66^{cd} (4.89)	31.31^{cd} (5.63)	54.32^{cd} (7.40)	65.67^{bc} (8.13)	19.32^{bc} (4.42)	29.33^{bc} (5.46)	52.33^{bc} (7.26)	56.67^{d} (7.56)	14.67^{cd} (3.88)	23.66^{bcd} (4.90)	45.67^{bc} (6.79)	54.67^{bcd} (7.42)	11.32^{cd} (3.43)	17.67^{bc} (4.25)	32.00^{bc} (5.68)	43.67^{abc} (6.63)
5	T5	30.00^{ab} (5.52)	39.66^{ab} (6.33)	63.6^{ab} (8.00)	72.33^{ab} (8.53)	24.31^{ab} (4.96)	35.31^{ab} (5.97)	59.31^{ab} (7.73)	69.32^{ab} (8.35)	21.67^{ab} (4.70)	29.32^{ab} (5.45)	53.67^{ab} (7.35)	61.32^{ab} (7.86)	16.00^{ab} (4.05)	24.66^{ab} (5.01)	38.67^{ab} (6.25)	48.32^{ab} (6.98)
6	T6	15.67^{e} (4.01)	24.67^{d} (5.01)	47.66^{d} (6.93)	54.67^{d} (7.42)	12.31^{d} (3.55)	21.33^{d} (4.65)	42.31^{d} (6.54)	48.31^{e} (6.97)	9.31^{e} (3.13)	16.32^{d} (4.09)	37.67^{d} (6.17)	47.67^{d} (6.93)	6.67^{e} (2.66)	11.31^{d} (3.44)	29.32^{c} (5.44)	35.32^{c} (5.97)
7	T7	1.00^{f} (1.17)	0.00^{f} (0.70)	0.00^{f} (0.70)	0.00^{f} (0.70)	0.00^{f} (0.70)	0.00^{f} (0.70)	0.00^{f} (0.70)	0.00^{g} (0.70)	0.00^{f} (0.70)	0.00^{f} (0.70)	0.00^{f} (0.70)	0.00^{f} (0.70)	0.00^{f} (0.70)	0.00^{f} (0.70)	0.00^{e} (0.70)	0.00^{e} (0.70)
8	T8	0.00^{f} (0.70)	3.00^{e} (1.72)	5.33^{e} (2.34)	7.67^{e} (2.80)	1.00^{e} (1.17)	3.00^{e} (1.72)	5.33^{e} (2.34)	7.67^{f} (2.80)	1.00^{f} (1.17)	3.00^{e} (1.72)	5.32^{e} (2.34)	7.67^{e} (2.80)	1.00^{f} (1.17)	4.00^{e} (1.73)	5.32^{d} (2.34)	7.67^{d} (2.80)
	CD	0.54	0.63	0.49	0.46	0.61	0.64	0.54	0.49	0.49	0.80	0.57	0.55	0.52	0.77	0.75	0.68
	SED	0.21	0.29	0.23	0.21	0.29	0.32	0.26	0.24	0.27	0.31	0.32	0.26	0.34	0.27	0.32	0.33

T_1 – *B. velezensis* VB7, T_2 - *B. hyensii*, T_3 – *B. velezensis*, T_4 – *B. subtilis,* T_5- *B. licheniformis*, T_6- *B.subtilis* T_7- LB broth (Control), T_8- Water (Control)

Figures in parentheses are square root transformed values

*Values are the mean of three replications
